# Three-dimensional a-Si/a-Ge radial heterojunction near-infrared photovoltaic detector

**DOI:** 10.1038/s41598-019-56374-2

**Published:** 2019-12-24

**Authors:** Xiaolin Sun, Ting Zhang, Linwei Yu, Ling Xu, Junzhuan Wang

**Affiliations:** 1 National Laboratory of Solid State Microstructures/School of Electronics Science and Engineering/Collaborative Innovation Center of Advanced Microstructures, Nanjing University, Nanjing, 210093 China; 2 Institute of Electronics Information Engineering, Sanjiang University, Nanjing, 210012 China

**Keywords:** Materials science, Nanoscience and technology

## Abstract

In this work, three-dimensional (3D) radial heterojunction photodetectors (PD) were constructed over vertical crystalline Si nanowires (SiNWs), with stacked hydrogenated amorphous germanium (a-Ge:H)/a-Si:H thin film layer as absorbers. The hetero absorber layer is designed to benefit from the type-II band alignment at the a-Ge/a-Si hetero-interface, which could help to enable an automated photo-carrier separation without exterior power supply. By inserting a carefully controlled a-Si passivation layer between the a-Ge:H layer and the p-type SiNWs, we demonstrate first a convenient fabrication of a new hetero a-Ge/a-Si structure operating as self-powered photodetectors (PD) in the near-infrared (NIR) range up to 900 nm, indicating a potential to serve as low cost, flexible and high performance radial junction sensing units for NIR imaging and PD applications.

## Introduction

Photodetectors (PDs) with a broad spectral response from ultraviolet (UV)-visible to near infrared (NIR) have attracted great attentions for a wide range of applications, which include imaging, thermo-sensing, communications, environmental monitoring and security surveillance^1–7^. As we know, the NIR and IR light can penetrate deeper into the bio-tissue, compared to the visible light, and thus have greater application potential in noninvasive biological detector and medical treatment^8–11^. So far, various materials such as III-V compounds and group IV photonics^12–16^ are frequently used in NIR PD application. Among them, Ge and related alloy PDs have an advantage as they can be fabricated in CMOS-compatible process for achieving a broader detection range^17–19^. Recently, there are also increasing research interests and efforts devoted to incorporating NIR PDs as photovoltaic bio-stimulator and detectors^20,21^.

Hydrogenated amorphous silicon (a-Si:H) thin film material has been widely used in constructing various optoelectronic devices, such as solar cells, because of its low-cost, low temperature and scalable fabrication process by plasma enhanced chemical vapor deposition (PECVD)^22–24^. However, the light absorption of a-Si:H is limited to the wavelengths <750 nm (due to its wider bandgap of ~1.7 eV), which is insufficient for NIR light detection. In comparison, hydrogenated amorphous germanium (a-Ge:H) with a narrower bandgap energy of ~1.1 eV^25^ can be used to expand the NIR absorption spectrum, while the deposition of a-Ge:H can also be accomplished via a low-temperature PECVD process^13,26^. Recently, there are many research efforts to expand the NIR absorption range by mixing a-Si:H and a-Ge:H to form a-SiGe:H alloy, which however suffer from a high defect density in the alloy with a low carrier mobility^27,28^. Furthermore, though the lattice quality of a-Ge:H can be improved by tuning deposition parameters^29^, a-Ge:H thin film alone is usually too defective to serve as high quality light absorbing layer, particularly for high performance PD applications^26,30,31^. Meanwhile, a-Si:H and a-Ge:H hetero-interface is known to be a Type-II band alignment, which can help to facilitate the separation of photo-generated carriers. Therefore, reducing the thickness of a-Ge:H absorber layer to facilitate carrier extraction, and adopting an a-Si:H passivation layer (PAL) at the heterojunction interface have been regarded as efficient ways to improve photovoltaic performances. For instances, D. Lundszien and T. Y. Lee *et al*. showed that a-Si:H thin film as a PAL at the p/i interface can increase open circuit voltage of solar cells^32,33^. D. P. Pham *et al*. reported that depositing a thin a-Si:H PAL at the p/a-SiGe:H interface can reduce the interface defects and significantly improve device performance^34^.

On the other hand, the reduced a-Ge:H layer thickness (and thus a reduced light absorption) has to be compensated by other structural design in order to achieve a sufficient light harvesting performance. To this end, three-dimensional (3D) radial junction (RJ) solar cells^35–38^, constructed over silicon nanowires (SiNWs), are particularly advantageous as they can achieve a strongly enhanced light trapping effect that allows for the use of a thinner absorber layer to achieve a fast carrier separation and collection. More importantly, the light-induced degradation problem can also be suppressed in the a-Si:H RJ solar cells with a rather thin absorber layer thickness <100 nm, as witnessed in our previous works^39–41^. In addition, it has been recently shown that the 3D RJ units can be directly constructed over flexible Al foil substrates with an excellent mechanical robustness against repetitive bending^42^, making them ideal candidates for flexible NIR bio-photoelectric stimulations or detections^43,44^.

Herein, we develop a new 3D SiNW architecture that integrates the PAL/a-Ge:H/a-Si:H light absorber layer in a 3D radial heterojunction (RHJ) structure to establish a high-performance NIR PD. The presence of ultra-thin a-Ge:H with only 6 nm thick can expand the absorption spectral of devices to a wider wavelength range spanning from 300 nm to 1050 nm. In parallel, the PAL at the p-SiNWs/a-Ge:H interface can not only modify the band gap but also reduce significantly the interface defect state density. By adjusting the thickness of the passivation layer, a rapid NIR photodetection is demonstrated with a fast response time of 2.62 μs in the NIR region.

## Method

### Device fabrication

**Figure 1:**
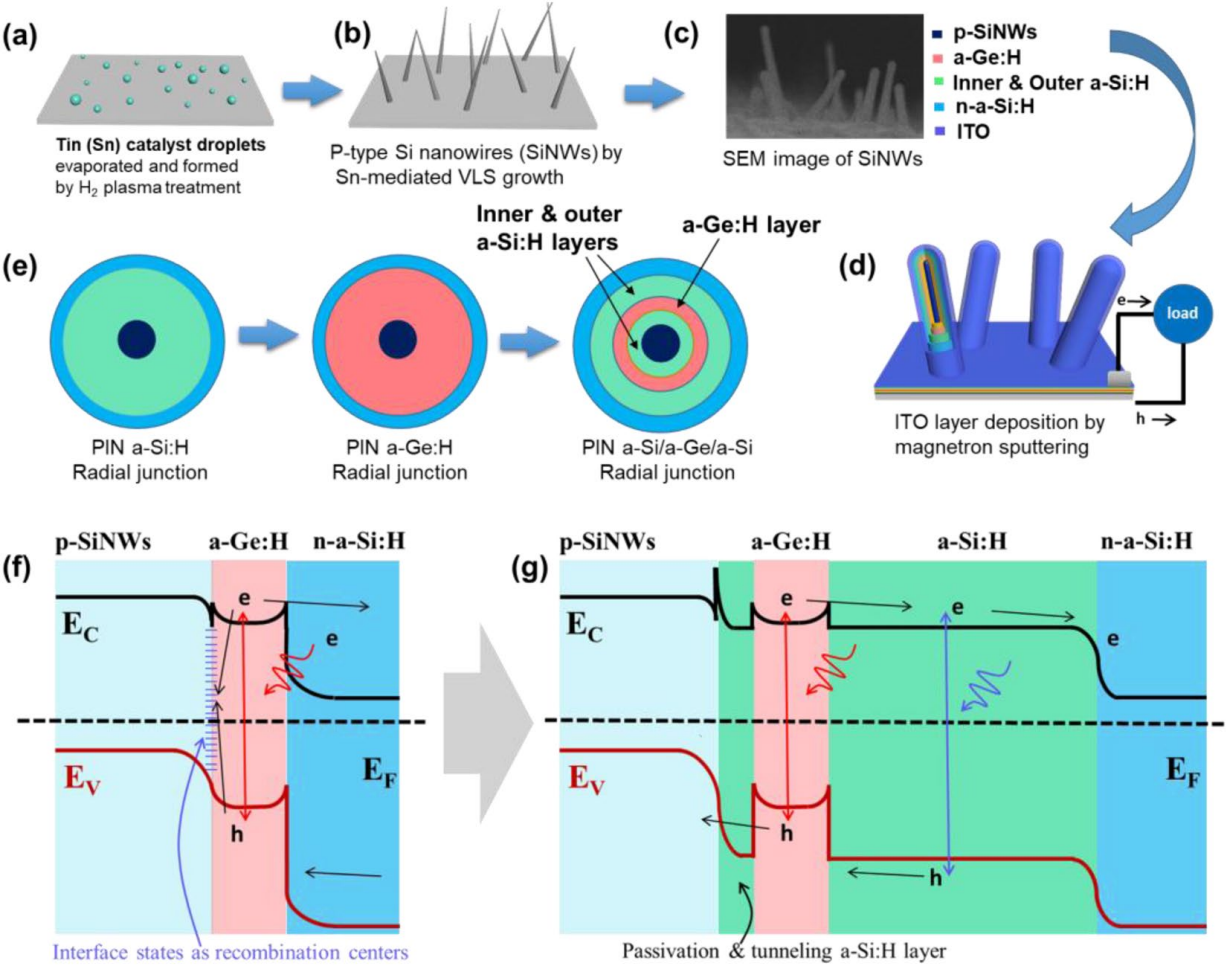
(**a**–**d**) Illustrate the fabrication procedures of PIN RHJ PAL/a-Ge:H/a-Si:H PD. (**e**) illustration of three types of Radial juntions: PIN a-Si:H RJ, PIN a-Ge:H RJ, PIN a-Si:H/a-Ge:H/a-Si:H RJ. (**f**) Sketches of the energy band profiles of PIN RHJ a-Ge:H PD where Ec is the conduction band and Ev is the valence band. (**g**) Sketch of PIN RHJ PAL/a-Ge:H/a-Si:H PD, showing the a-Ge:H quantum well confined by the a-Si:H shell.

Fabrication of PIN RJ PDs: Before evaporating a thin tin (Sn) layer of 2 nm, the AZO (Al-doped ZnO, bottom electrode) glasses were cleaned in acetone, methanol and deionized water by ultrasonication. Then, the Sn layer was treated in PECVD system by H_2_ plasma for 5 min to form discrete Sn droplets for subsequent catalyzing vertical SiNWs upon the AZO surface. Then, p-type SiNWs with a typical length of 1 μm and a mean diameter of 30 nm-40 nm were formed via VLS model, with the introduction of silane (SiH_4_) and diborane dopant (B_2_H_6_) gases at 400 °C. In the next step, intrinsic a-Si:H absorber and n-type a-Si:H layers, with the thicknesses of around 80 nm and 10 nm were subsequently deposited upon the SiNWs cores at 150 °C, respectively. Finally, a transparent ITO layer (~70 nm) as the top electrode was deposited by magnetron sputtering (140 W, 700 s) around the RJ units. The transverse cross-sectional top-view was shown in Fig. 1e, PIN a-Si:H Radial junction.

Fabrication of RHJ a-Ge:H PDs: The process of SiNWs growth, n-type a-Si:H and ITO deposition processes were the same as PIN RJ solar cells, where the intrinsic a-Si:H absorber layer was replaced by a-Ge:H layer with the growth gas of Germane (GeH_4_). The transverse cross-sectional view was shown in Fig. 1e, PIN a-Ge:H Radial junction.

Fabrication of RHJ PAL/a-Ge:H/a-Si:H PDs: The process of SiNWs growth, n-type a-Si:H and ITO deposition were same as PIN RJ solar cell. The intrinsic absorber includes three layers: a-Si:H PAL, a-Ge:H layer and outer a-Si:H layer. The thickness of PAL (~35 nm, ~23 nm, ~12 nm, 0 nm) was changed with deposition time (30 min, 20 min, 10 min, 0 min). The thickness of a-Ge:H layer (~16 nm, ~6 nm, ~3 nm, 0 nm) was changed with deposition time (30 min, 10 min, 5 min, 0 min). The cross-sectional view was illustrated in Fig. 1e, PIN a-Si:H/a-Ge:H/a-Si:H Radial junction, the corresponding production process was shown in Fig. 1a–d.

### Device characterizations

The Current Density-Voltage (J-V) Characterization: The J-V characteristics were measured with standard AM1.5 G illumination (Newport, Oriel Sol-1A), where the external quantum efficiency (EQE) was measured by QEX-10 measurement System, with a sweep step of 10 nm from 300 nm to 1100 nm. We manufactured mask with several 1.5 mm × 4 mm rectangular patterns on it, and electrode ITO was only deposited in the rectangular region during magnetron sputtering. As for the impact of beam spot size and position, the EQE measurement has been carried out with a spot size (~1.8 mm × 4 mm) that can cover around 80% of the ITO electrode area.

Response Speed Characterization: NIR Monochromatic (λ = 808 nm) spectrum was produced by a pulsed laser (MDL-H-808nm-4W-17091506). The frequencies of light source were modulated ranging from 5 Hz to 50 kHz. The response results were displayed through a Keysight DSOX3052A oscilloscope.

## Results and Discussion

**Figure 2:**
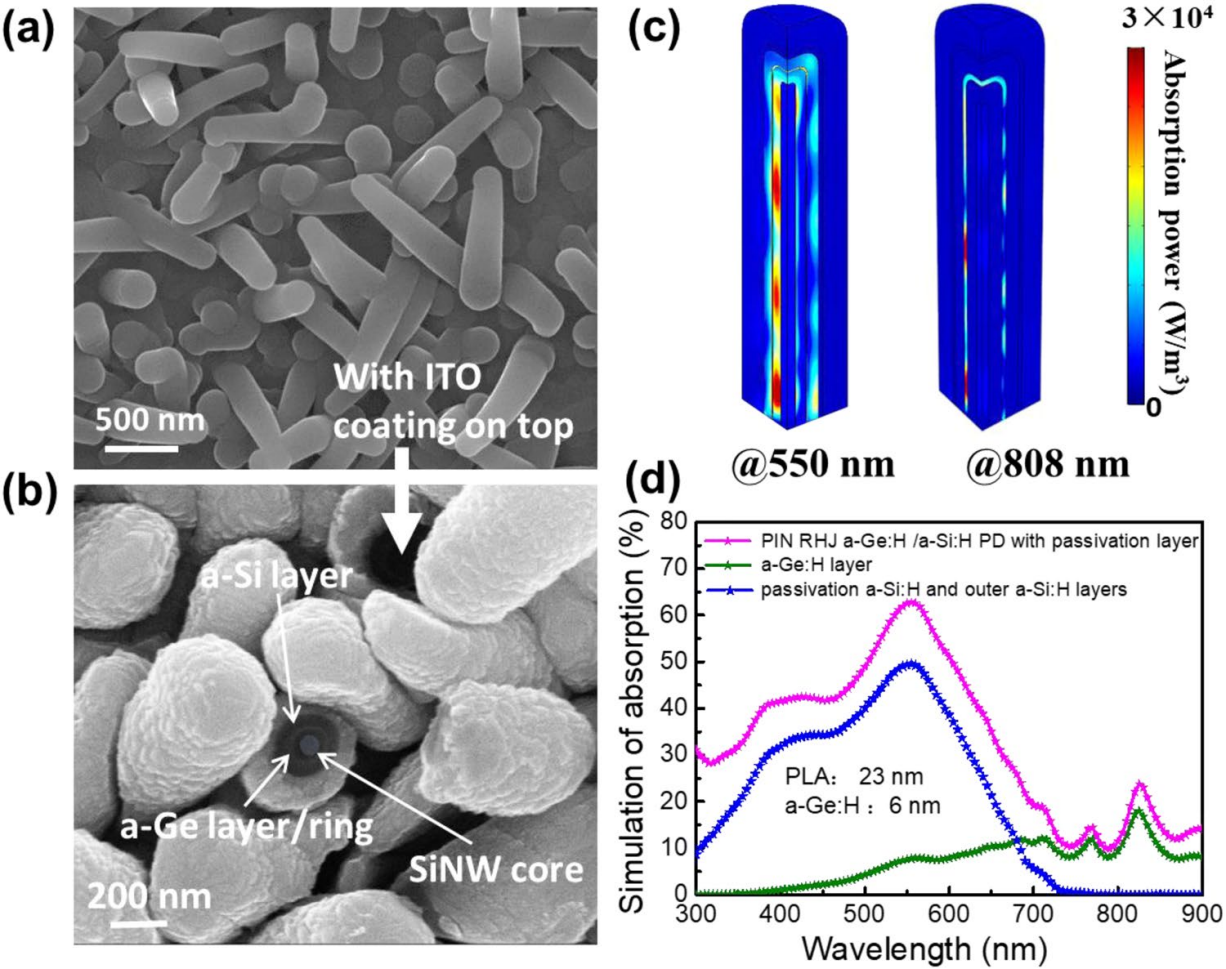
(**a**,**b**) Illustration of the SEM images of the PIN RHJ PAL/a-Ge:H/a-Si:H PD, before and after ITO coating, the inset shows the RHJ unit in full details, the corresponding finite element simulated absorption profile within the unit at 550 nm, 808 nm simulated by COMSOL shows in (**c**), the simulation of absorption extracted from RHJ unit show in (**d**).

A typical SEM image of a randomly oriented of PIN RHJ PAL/a-Ge:H/a-Si:H heterojunction is shown in Fig. 2a. It is interesting to note that, while the multilayer thin film coating by PECVD can be quite conformal around the SiNWs, the deposition of ITO by magnetron sputtering is not so uniform, especially along the depth of standing RJ units, as shown in Fig. 2b. Among the random but mutual-crossed RJ units, there exist extra current paths, which can help to transport and collect the photo-current among the crossed RJs, that has been analyzed in our previous work^42^.

**Figure 3:**
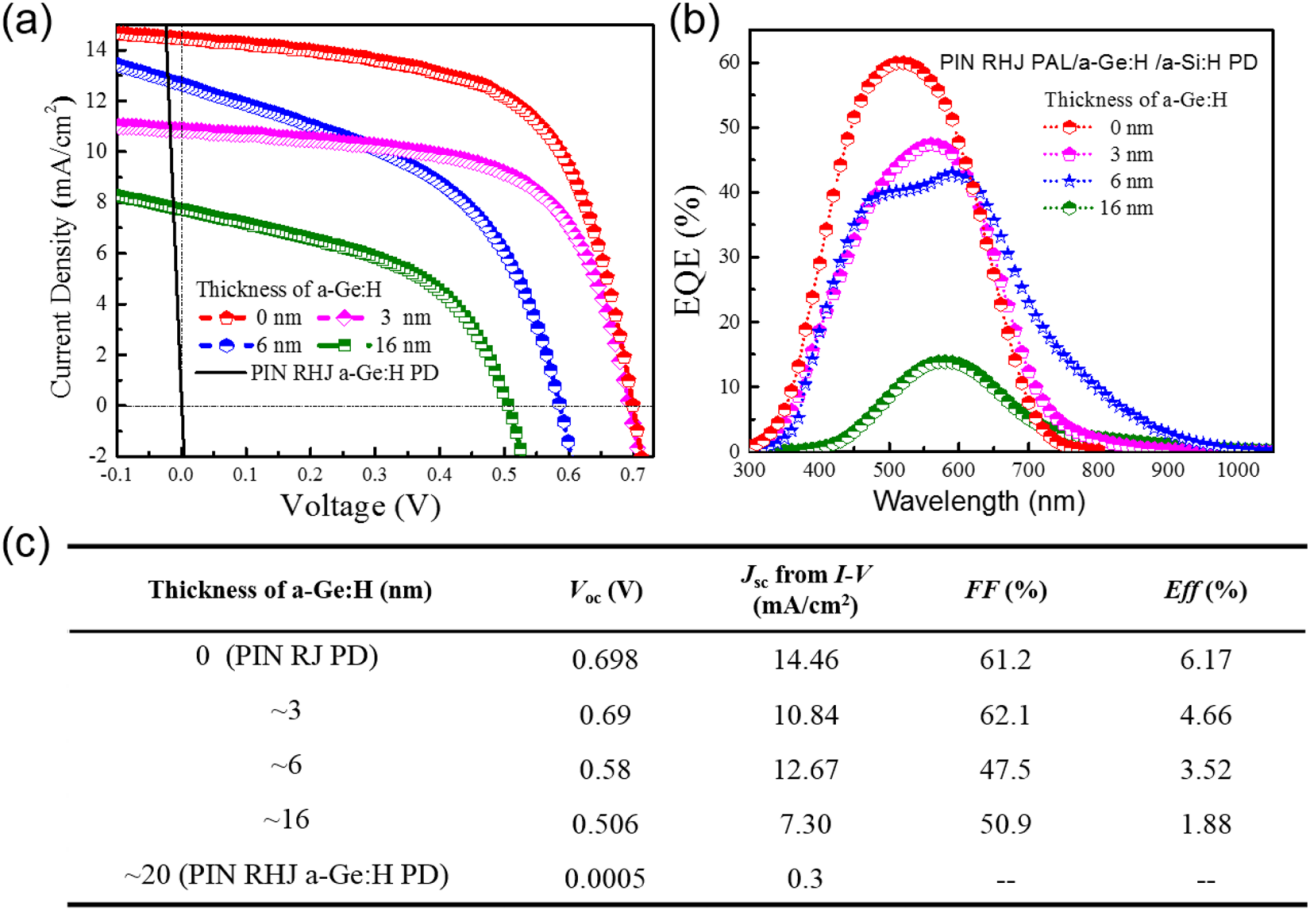
(**a**–**c**) Show the current density-voltage (J-V), EQE curves and performance parameters of PIN RHJ PAL/a-Ge:H/a-Si:H PD with different a-Ge:H thicknesses when fixed the PAL thickness at ~35 nm.

First and foremost it is worth noting that the *J-V* characteristics and performance parameters of PIN RJ PD (corresponding to the device with a-Ge:H film thickness equals to 0 nm) were presented in Fig. 3a,c respectively. A short circuit current density (*J*_*sc*_) of ~14.46 mA/cm^2^, open circuit voltage (*V*_*oc*_) of ~698 mV, fill factor (FF) of ~61%, and power conversion efficiency (PCE) of ~6.2% were obtained for the PIN RJ PD. While as shown in Fig. 3b, the EQE of PIN RJ PD is almost zero with the wavelength longer than 800 nm, indicating extremely weak or no absorption of the NIR due to the large bandgap of a-Si:H. In order to extend the absorption range to the NIR, we fabricated PIN RHJ a-Ge:H PD with a single a-Ge:H absorber layer (named as PIN RJ a-Ge:H PD). As shown in Fig. 3a,c, the performance of the device is extremely poor, almost has no photovoltaic response. The main causes are related to these factors: there is defect-rich region in the a-Ge:H film as well as in p-SiNWs/a-Ge:H interface, what’s more, the valence band offset at the p/i interface where there is a trap for holes will degrade forward diffusion of holes (illustrated in Fig. 1f)^29^.

In order to passivate the interface states as well as to improve the band energy alignment, we added an a-Si:H thin film as a PAL between p-SiNWs and a-Ge:H film and optimized the thickness of a-Ge:H film (~16 nm, ~6 nm and ~3 nm) to get PIN RHJ PAL/a-Ge:H/a-Si:H PD. Compared with the PIN RHJ a-Ge:H PD, the *J-V* characteristics and EQE response of RHJ PAL/a-Ge:H/a-Si:H PD are obviously improved, as shown in Fig. 3a–c. The optimal thickness of a-Ge:H film is 6 nm, corresponding EQE response reaching ~10% at the wavelength of 808 nm, and *J*_*sc*_ still maintaining 12.67 mA/cm^2^. We attributed the improvement to that PAL decreases the interface defect density as well as acts as an electron blocking layer, which clearly presented in the energy band diagram shown in Fig. 1g. However, considerable deterioration of *J-V* characteristics and EQE response in infrared region were caused when the thickness of a-Ge:H film is thicker (16 nm) or thinner (3 nm), which mainly because there exits high defect state density in a-Ge:H film when it is too thick and the infrared absorption is insufficient when it is too thin. It is noticeable that better EQE response was obtained in near infrared region (800 nm~1050 nm) for PIN RHJ PAL/a-Ge:H/a-Si:H PD with 6 nm ultrathin a-Ge:H film.

**Figure 4:**
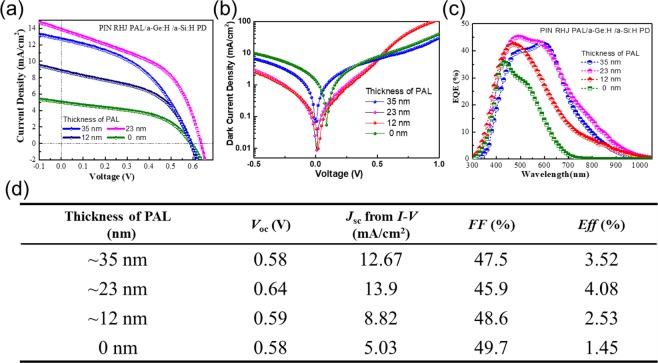
(**a**–**d**) Show the current density-voltage (J-V), IV dark characteristics, EQE curves and performance parameters of PIN RHJ PAL/a-Ge:H/a-Si:H PDs with different PAL thicknesses when fixed the a-Ge:H thickness at ~6 nm.

We also analyzed the relationship between the thickness of PAL and NIR detection performance, as shown in Fig. 4a–c. The *J-V* characteristics and EQE response changed significantly with various thicknesses of PAL. Remarkably, when the thickness of PAL is of ~23 nm, the EQE can reach ~15% at the wavelength of 808 nm (Fig. 4b), corresponding *J*_*sc*_ of 13.9 mA/cm^2^ and *V*_*oc*_ of 640 mV (shown in Fig. 4c), which are almost as better as that of the PIN RJ PD.

As shown in Fig. 4a–d, the thickness of PAL can affect the electrical characteristics and spectral characteristic of PIN RHJ PAL/a-Ge:H/a-Si:H PD significantly. This is mainly because a-Ge:H layer will absorb photons under near-infrared radiation and photo-generated electrons will drift easily from a-Ge:H film to n-a-Si:H layer under the built-in electric field. However, photo-generated holes will be trapped in the region of the a-Ge:H layer because of the hole well, as shown in Fig. 1g, and difficult to transport to p-SiNWs. Therefore, if the PAL gets too thick (~35 nm), the photo-generated holes will accumulate in the a-Ge:H region and hard to be collected by the inner electrode, If the PAL gets too thin (~12 nm), there will exist high defects density at the interface of a-Ge:H/p-SiNWs. Both of them will result in the decrease of photocurrent. Furthermore, as shown in Fig. 4b, by introducing a 23 nm thick PAL, a significant reduction in the dark-current to 10 nA at 0 V reverse bias is observed, This dark current is ~one order of magnitude less than the PDs fabricated without this PAL.

Therefore, only when the thickness of PAL is appropriate (~23 nm), the photo-generated holes in a-Ge:H region might reach the p-SiNWs by tunneling effect, thus improving the performance of PD.

Meanwhile, the light distributions of devices under incident light (550 nm and 808 nm) are simulated by COMSOL to reveal the underlying reasons for high NIR absorption performance of PIN RHJ PAL/a-Ge:H/a-Si:H PD. As seen in Fig. 2c, a strong light absorption was observed under the visible irradiation (550 nm) in the multi absorber layer. Noticeably, a weak light absorption can be found in ultrathin (less than 10 nm) a-Ge:H film under 808 nm, which suggests that the a-Ge:H layer is very sensitive to NIR. Furthermore, the simulation of absorption of PAL, outer a-Si:H layer and a-Ge:H layer are presented in Fig. 2d. We can find that the outer a-Si:H layer and PAL absorb well in the visible region, while a-Ge:H layer mainly captures infrared light. The absorption of the device reaches about 15% at 808 nm wavelength, which is close to the actually measured EQE of RHJ PAL/a-Ge:H/a-Si:H PD.

**Figure 5:**
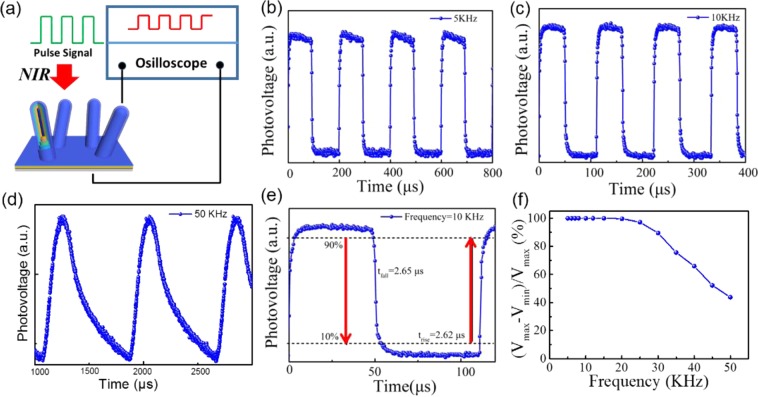
(**a**) Schematic illustration of RHJ PAL/a-Ge:H/a-Si:H PDs under pulsed NIR light irradiation (808 nm). Photoresponse of device under different frequencies: (**b**) 5 kHz, (**c**) 10 kHz, (**d**) 50 kHz. (**e**) The corresponding magnified plots of one response cycle @10 kHz at 808 nm wavelength. (**f**) Relative balance (Vmax-Vmin)/Vmax versus switching frequency from 5 kHz to 50 kHz.

Response speed as one of the most import parameters reflects the carrier separation and recombination in the devices, which is widely used in the optical imaging and communication. To evaluate the response time, we employ a function generator equipped with an illumination light (808 nm, schematically illustrated in Fig. 5a), which can generate different frequencies (from 5 kHz to 50 kHz). As shown in Fig. 5b–d, the device shows a stable and reproducible response oscillogram at the frequencies of 5 kHz, 10 kHz and 50 kHz. The rise time (t_rise_) and fall time (t_fall_) were extracted from the one cycle at 10 kHz with a value of 2.62/2.65 μs as presented in Fig. 5e, which is faster than the previous reported NIR PDs based on TMD, quantum-dot materials. A cutoff frequency (*f*_3dB_) of 3 dB bandwidth of 3D PIN RHJ Sandwich structure NIR PD can be determined by the following equation: *f*_3dB_ = 0.35/t_rise_, and the corresponding value of device is estimated to be 133 kHz. Further investigation of photovoltage as a function of frequency are depicted in Fig. 5f in which the relative of (V_max_ − V_min_)/V_max_ only decreases 11% at ultra-high pulse frequency of 30 kHz, which confirms that the 3D PIN RJ sandwich structure NIR PD shows a stability in the signal output and can work at an even higher frequency.
